# Cord blood-derived CD19-specific chimeric antigen receptor T cells: an off-the-shelf promising therapeutic option for treatment of diffuse large B-cell lymphoma

**DOI:** 10.3389/fimmu.2023.1139482

**Published:** 2023-06-27

**Authors:** Tiantian Yu, Cancan Luo, Huihui Zhang, Yi Tan, Li Yu

**Affiliations:** ^1^Department of Hematology, The Second Affiliated Hospital of Nanchang University, Nanchang, Jiangxi, China; ^2^Division of Hematopathology and Department of Pathology, Duke University Medical Center, Durham, NC, United States; ^3^R&D Department, Qilu Cell Therapy Technology Co., Ltd., Jinan, Shandong, China

**Keywords:** cancer immunotherapy, chimeric antigen receptor T cells, CD19, cord blood, diffuse large B-cell lymphoma

## Abstract

**Purpose:**

Autologous chimeric antigen receptor (CAR) T cell therapy is one of the most significant breakthroughs in hematological malignancies. However, a three-week manufacturing cycle and ineffective T cell dysfunction in some patients hinder the widespread application of auto-CAR T cell therapy. Studies suggest that cord blood (CB), with its unique biological properties, could be an optimal source for CAR T cells, providing a product with ‘off-the-shelf’ availability. Therefore, exploring the potential of CB as an immunotherapeutic agent is essential for understanding and promoting the further use of CAR T cell therapy.

**Experimental design:**

We used CB to generate CB-derived CD19-targeting CAR T (CB CD19-CAR T) cells. We assessed the anti-tumor capacity of CB CD19-CAR T cells to kill diffuse large B cell lymphoma (DLBCL) *in vitro* and *in vivo*.

**Results:**

CB CD19-CAR T cells showed the target-specific killing of CD19+ T cell lymphoma cell line BV173 and CD19+ DLBCL cell line SUDHL-4, activated various effector functions, and inhibited tumor progression in a mouse (BALB/c nude) model. However, some exhaustion-associated genes were involved in off-tumor cytotoxicity towards activated lymphocytes. Gene expression profiles confirmed increased chemokines/chemokine receptors and exhaustion genes in CB CD19-CAR T cells upon tumor stimulation compared to CB T cells. They indicated inherent changes in the associated signaling pathways in the constructed CB CAR T cells and targeted tumor processes.

**Conclusion:**

CB CD19-CAR T cells represent a promising therapeutic strategy for treating DLBCL. The unique biological properties and high availability of CB CD19-CAR T cells make this approach feasible.

## Introduction

One of the developmental milestones in immunotherapy of hematologic malignancies is chimeric antigen receptor (CAR) T cell therapy (1). Genetically engineered T cells expressing CARs can specifically target tumor cells (2). CAR is a fusion protein consisting of an extracellular domain binding target antigen and linked to an intracellular signaling domain. First-generation CARs were designed using only the CD3ζ intracellular signaling domain of the TCR/CD3 complex. Second- and third-generation CARs contain costimulatory molecules fused to CD3ζ, such as CD28 and/or 4‐1BB, which leads to enhanced proliferation, durable activity, cytokine secretion, apoptotic resistance, and *in vivo* persistence (2). Currently, the Food and Drug Administration has approved the use of four CAR T programs as third-line therapy of large B cell lymphoma (LBCL): BREYANZI (lisocabtagene maraleucel) (3), Novartis’s KYMRIAH (tisagenlecleucel) (4), Gilead’s YESCARTA (axicabtagene ciloleucel) (5), and Gilead’s TECARTUS (brexucabtagene autoleucel) (6) and second-line therapy of LBCL: YESCARTA (7). The overall response rate has been observed to be as high as 73% with 54% complete response (CR) rate (8). With this clinical success, CAR T cells have revolutionized the treatment of relapsed/refractory (R/R) LBCL.

Use of autologous CAR T (auto-CAR T) cells targeting CD19 has led to outstanding data for patients with R/R LBCL (9). However, following leukapheresis, auto-CAR T cell engineering is a bespoke fabrication procedures for all patients, leading to certain well-known shortcomings, such as high out-of-pocket payments and prolonged wait time. Some patients may show disease progression or may lose eligibility for treatment-related complications over the waiting period, causing delayed or failed availability of auto-CAR T cell therapy (10). Moreover, auto-CAR T cells may be ineffective owing to T cell dysfunction, wherein immunosuppression receptors are expressed (11). The functional characteristics of auto-CAR T cells are inversely affected by the previous accumulation effects of chemotherapy (12). For these reasons, some patients fail to receive autologous T cells for producing CAR T cell products (13). Finally, the cost of this auto-CAR T cell therapeutic approach remains high and it is not readily available for all patients, which is a challenge for healthcare systems (14).

The ‘off-the-shelf’ allogeneic CAR T (allo-CAR T) cells from healthy donors with simplified and standardized manufacturing are expected to address these problems. Allo-CAR T cells host several prospective advantages, for example lower and affordable costs, owing to the application of scaled manufacturing processes and the capacity to generate multiple CAR T cells from a single donor (15). Allo-CAR T cells with pre-prepared and cryopreserved features can be taken as needed, making therapy available instantly for patients (15). In addition, a crucial difference is that allogeneic cell manufacturing involves a batch of products, which can be used if repetition is necessary. In contrast, a collection of autologous cells can only be used to produce a single-cell product. The ‘off-the-shelf’ allo-CAR T cells also can combinate with antibody targeting co-inhibitory molecule (16). Clinical studies have shown that donor-derived CAR T cells exhibit effective expansion in patients with acute lymphoblastic leukemia (ALL), achieving a high CR and controllable safety (17). However, allogeneic approaches suffer from two significant problems. First, allogeneic T cells may lead to life-threatening graft-versus-host disease (GVHD). Second, the host immune system may rapidly recognize and eradicate allogeneic T cells, thereby limiting their anti-tumor activity (18).

Allo-CAR T cells are primarily derived from peripheral blood mononuclear cells (PBMCs) and not often from cord blood (CB). CB transplantation has been successfully used to cure hematologic malignancies in recent decades, owing to decreased graft failure rates and transplantation-related mortality. Research indicates that the exceptional biological characteristics of CB cells may result in improved anti-cancer efficacy. Therefore, CB could be an ideal option for immunotherapy, offering products that are readily accessible ‘off-the-shelf’ (19). CB-derived CAR-NK cell therapy has been successfully used to treat hematologic malignancies. 73% (8/11) of patients responded to treatment with CB-derived CARNK cells without developing major toxic effects (20). Through genetic manipulation and stimulation of costimulatory molecules, the formerly naïve CB T-cell has been directed to differentiate into effector T cells (21). In a mouse model of ALL, CB-derived CAR T cells show a higher naïve T cells proportion and better tumor growth inhibition than PB-derived CAR T cells from R/R ALL patients (22). However, the number of clinical trials using CB-derived CAR T cells products is limited. Thus, we generated CB-derived CD19-targeting CAR T cells and assessed the anti-tumor activity of CB CD19-CAR T cells in diffuse large B cell lymphoma (DLBCL).

## Materials and methods

### Cell lines, cell culture, and animal experiments

SUDHL-4, DB, BV173, and K562 cells were obtained from the Stem Cell Bank of the Chinese Academy of Sciences. All cell lines were cultured in RPMI-1640 medium supplemented with 10% fetal bovine serum (Gibco, Billings, MT, USA). All cell lines were authenticated by Short Tandem Repeat profiling and regularly tested negative for mycoplasma contamination.

This study used male BALB/c nude (BALB/c-nu) mice aged 5-6 weeks, purchased from Hunan Slake Jingda Experimental Co., Ltd. Ethical approval was received from the Medical Research Ethics Committee of the Second Affiliated Hospital of Nanchang University, and written informed consent was obtained. A total of 1 × 10^6^ SUDHL-4 cells suspended in a mixture of 100 μL Matrigel and PBS were subcutaneously injected into the backs of BALB/c-nu immunodeficient mice. Definition tumor engraftment at day 9, mice were then randomly divided and received CB CD19-CAR T cells in treatment groups and CB T cells in control groups at day 10, and tumor measurement was monitored every 3 days (n=7 per group). After experimental observation, the mice were euthanatized following the painless cervical dislocation, and their tumors were collected for subsequent analyses.

### CD19-CAR construct design and lentiviral vector production

The construct of generating the CD19 CAR lentiviral was performed based on the methods previously published in a patent (CN 108753774 B). The scFv (VL-linker-VH) sequence of CD19 CAR was encoded using synthetic DNA technology (GENEWIZ, China). Next, the CAR was subcloned into a second generation with a 4-1BB costimulatory domain. A truncated version of the CD19 CAR was created by deleting the cytoplasmic domains. 293T cells transfected with packaging plasmids and the scFv vector, including CAR construct, generate lentiviruses products. The viral supernatant was harvested after 48–72h, concentrated and stored at −196°C until further use.

### T-cell isolation, culture, and transduction

According to the manufacturer’s instructions, we isolated CD3+ T cells from fresh cord blood by CD3 positive selection microbeads (Miltenyi Biotech, Germany). For activating the T cells, we resuspended the isolated CD3+ T cells (1 × 10^6^ cells/ml) in X-VIVO 15 medium (Thermo Fisher Scientific, Waltham, MA, USA) supplemented with 5% human AB serum (Thermo Fisher Scientific) and 200 U/mL recombinant human IL-2 (PeproTech, USA) at 37°C in 5% CO_2_. Sterile, non-tissue-culture-treated 24-well plates were coated with Retronectin (Thermo Fisher Scientific) at 6 µg/cm^2^ and left to stand overnight at 37°C in 5% CO_2_. Next, the lentivirus supernatant was transferred to plates, and then T cells activated using recombinant human interleukin-2 (250 U/mL) were added, followed by incubation at 37°C for 24h after centrifugation. The medium was changed 24h later and every other day afterwards.

### Cytotoxicity and multiplex cytokine assay

All anti-human antibodies, including CD45RA-APC (Cat: 550855), CD3-FITC (Cat: 555339), CD4-APC-Cy7 (Cat: 557871), CD8-PerPCy5.5 (Cat: 560662), CCR7-PE (Cat: 552176), CD27-PE-Cy7 (Cat: 560609), CD28-BV711 (Cat: 563131), Fixable Viability Stain (FVS) (Cat: 562247), PD-1-BV421 (Cat: 562584), and TIM-3-BV605 (Cat: 747961), were purchased from BD Pharmingen (BD Biosciences, Franklin Lakes, NJ, USA). Tumor cells were labeled with 2 µM intracellular tracing reagent carboxyfluorescein succinimidyl ester (CFSE) (Invitrogen, Waltham, MA, USA), and dead cells were marked with FVS. All flow cytometric analyses were performed using a BD FACSCanto (BD Biosciences) and analyzed with FlowJo Version 10 (Tree Star, Ashland, OR, USA). The capacity of CB CD19-CAR T cells recognizing and killing target cells was evaluated by analyzing the percentage of CFSE-labelled target cells after coculturing for 24 h at different effector: target (E: T) ratios of 1:1, 2:1, 5:1, 10:1. Supernatants were harvested after 48 h, and multiple cytokines (IL-2, IL-4, IL-6, IL-10, TNF-α, and IFN-γ) were detected using the BD Cytometric Bead Array (CBA) Human Th1/Th2 Cytokine Kit (BD Pharmingen) by flow cytometry.

### RNA sequencing analysis

CB T and CB CD19-CAR T cells were collected (three biological replicates) as samples for RNA-seq analysis. This RNA-seq was also used to analyze CB CD19-CAR T cells before and after co-culture with SUDHL-4 cells an E: T ratio of 1:1 for 48h. cDNA library construction, library purification, and transcriptome sequencing were executed using the DNBseq platform according to the instructions provided by Kindstar Global Company (www.kindstar.com.cn). For RNA-seq data, the gene expression levels were quantified in fragments per kb of exon model per million mapped reads exon model. The differentially expressed genes (DEGs) were analyzed using EdgeR software and the significance was adjusted *P*-value of <0.05 and absolute log^2^ (absolute ratio value) ≥ 1.

### Statistical analyses

Statistical analyses were executed using GraphPad Prism 8.0 (GraphPad Software). Two-tailed Student’s *t*-test was used to compare two groups to identify significant differences. A two-way ANOVA with Tukey’s multiple comparison test was used for three and more groups. For experiments in the animal tumor model, two-way ANOVA was used to analyze tumor volume and weight. Experimental data were collected from a minimum of three independent experiments for each analysis. Data are presented as the mean ± standard error of means (SEM), and statistical significance was set at *P* < 0.05.

### Data availability

The data generated in this study are available upon request from the corresponding author. Data were generated by the authors and included in the article

## Results

### Generation and characterization of CB CD19-CAR T cells

**Figure 1:**
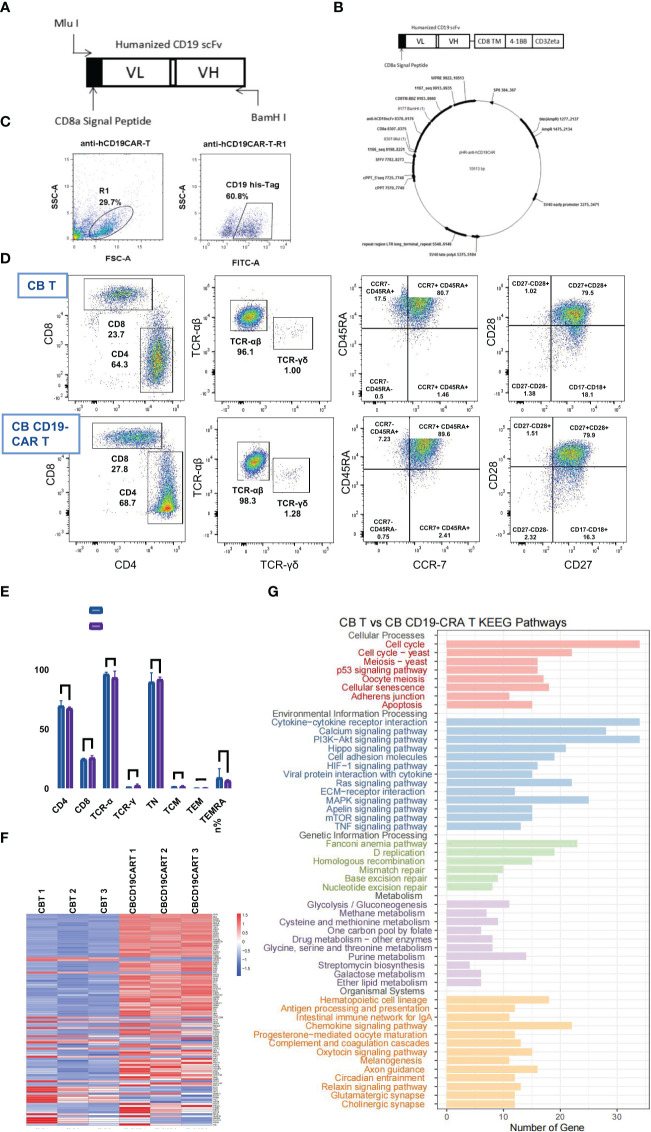
The construction, characterization, and gene expression of CB CD19-CAR T. **(A)** Schematic diagram of anti-hCD19 scFv. **(B)** Schematic diagram of plasmid construct for pHR- anti-hCD19CAR. **(C)** Representative flow cytometry analysis of transduction efficiency of CB T cells. **(D)** Representative flow cytometry analysis of the maturation profile shows there is no difference found in either the fraction of CB CD19-CAR T cells or CB T cells. **(E)** Data show mean ± SEM. **(F)** The heatmap of top 50 DEGs expression profiles. **(G)** KEGG pathway functional enrichment analyses of CB CD19-CAR T cells compared with CB T cells. TN, Naive T cell; TCM, Central memory T cell; TEM, effector memory T cell; TEMRA, Terminal effector T cell; DGEs, differentially expressed genes; SEM, standard error of means; ns, non-significant.

We generated a CD19scFv-based CAR construct with a 4-1BB costimulatory domain and a CD3ζ signaling domain (**Figures 1A, B**). CB T cells without CD19 CAR transduction were used as control. Activated CB T cells were transfected with lentiviral vectors, with consequential expression of CD19 CAR (**Figure 1C**). Subsequently, we tested the immunophenotypes of CB CD19-CAR T and CB T. Flow cytometry data indicated that the proportions of CD4, CD8, TCR-α, and TCR-γ cells did not differ between CB CD19-CAR T and CB T (**Figures 1D, E**). Following expansion, both CB CD19-CAR T and CB T cells were enriched for naïve T cells (CD45RA+CCR7+; CD28+CD27+), indicating that they were similarly cultured with no significant proliferation differences. We performed RNA*-*seq analysis of CB CD19-CAR and CB T cells (**Figures 1F, G**). CB CD19-CAR T cells manifested changes in genes related to adherens junction, cytokine-cytokine receptor interaction, chemokine signaling pathway and antigen processing and presentation. This may be associated with the assembly of CAR to enhance cell membrane’s function and T cell immune response.

### CB CD19-CAR T cells specifically recognize and kill BV173 cells

**Figure 2:**
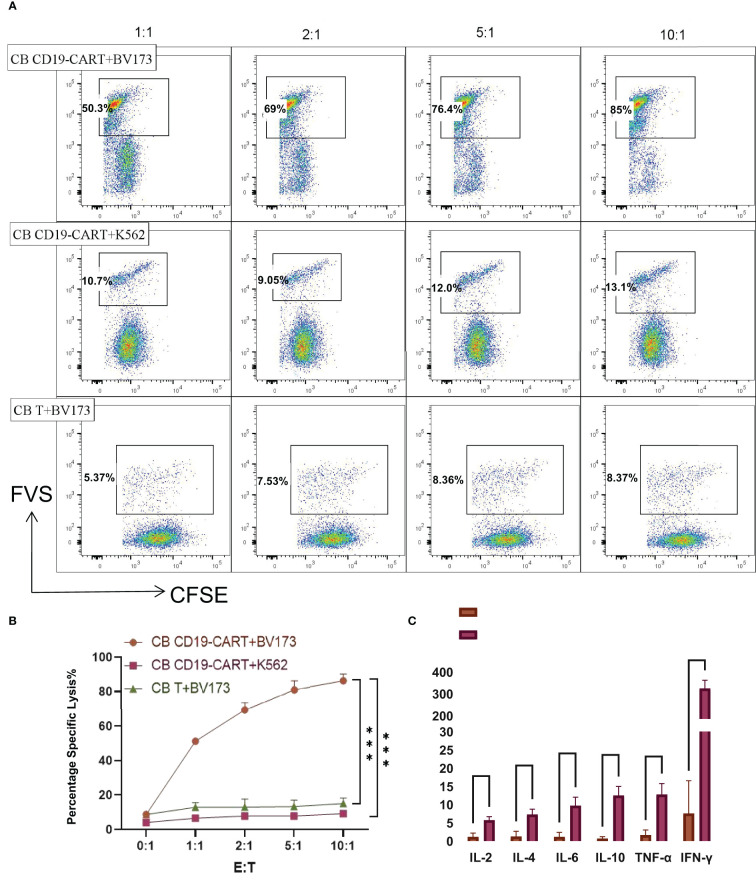
CB CD19-CART specifically kills CD19+ BV173 cells. **(A)** Representative dot plots of cytotoxicity assays showing specific on-target killing. The CFSE+FVS+/CFSE+ ratio was used to determine the kill rate. **(B)** Data show mean ± SEM of specific cytotoxicity experiments. **(C)** Statistical diagram of cytokine concentration in CB T or CB-CD19-CAR T cells cocultured with BV173 cells at an E: T ratio of 1:1 for 24h. SEM, standard error of means; ns, non-significant; ***P < 0.001, ****P < 0.0001.

To investigate the cytolytic ability of CB CD19-CAR T cells for distinguishing and eliminating CD19+ tumor cells, we first selected BV173 cells (a CD19+ ALL cell line) for verification. Compared with the CB T group and CD19-negative cell line K562 group, CB CD19-CAR T cells mediated cytotoxicity against the CFSE-labelled BV173 cells (*P* < 0.05, n = 3; **Figures 2A, B**), indicating that CB T cells expressing CAR constructs were able to eliminate tumor cell. We also detected cytokine products of CB CD19-CAR T cells following coculture with target tumor cells for the examination of the effector function. Supernatants analyzed by the CBA assay revealed that only BV173 group could elicit release of multiple cytokines by CB CD19-CAR T cells (**Figure 2C** and **Figure S1A**), further indicating that the CB CD19-CAR T cells exhibited specific activation with target cell stimulation.

### CB CD19-CAR T has potent anti-tumor efficacy against CD19+ DLBCL cells *in vitro* and *in vivo*

**Figure 3:**
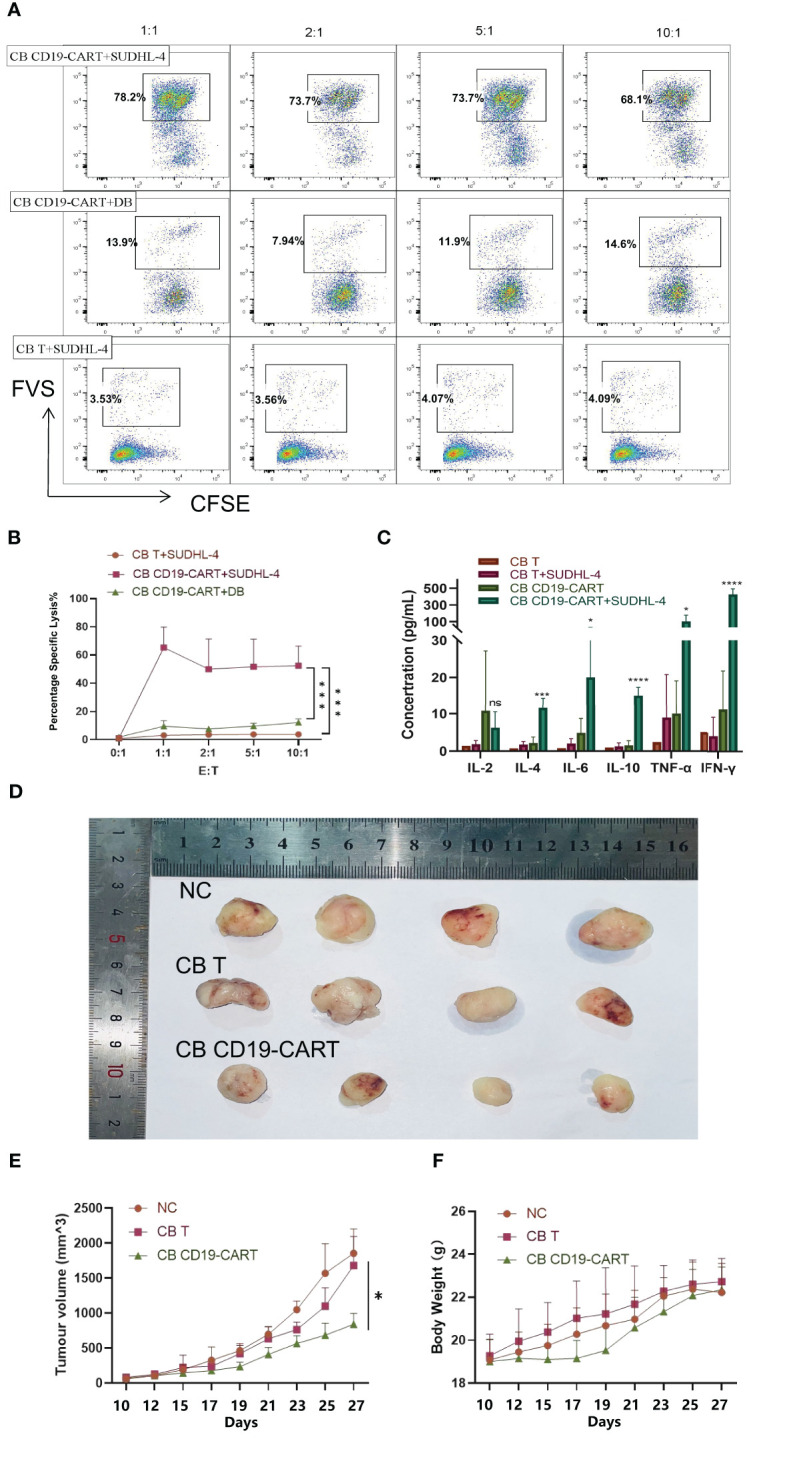
Specific cytotoxicity of CB CD19-CAR T cells targeting CD19+ DLBCL cells. **(A)** Representative plots showing specific cytotoxicity of CB CD19-CAR T cells against CD19+ DLBCL cells but not CD19- DLBCL cells. **(B)** Quantitative data of the cytotoxic activity of CB-CD19-CAR T cells and CB T cells against DLBCL cell lines. Error bars represent SEM. **(C)** Cytokine concentration in CB T or CB-CD19-CAR T cells cocultured with SUDHL-4 cells at an E: T ratio of 1:1 for 24h. **(D)** Representative tumor resectates from each group. **(E, F)** Data are expressed as mean ± SEM of tumor masses and body weight (n=4 mice per group). SEM, standard error of means; ns, non-significant; *P < 0.05; ***P < 0.001; ****P < 0.0001.

SUDHL-4 cells are DLBCL cells expressing CD19 markers that can be recognized explicitly by CB CD19-CAR T cells. DB cells are DLBCL cells that are not CD19-positive. To confirm their cytotoxicity against CD19+DLBCL cells, we cocultured SUDHL-4 and DB cells with CB T and CB CD19-CAR T cells at different E: T ratios of 1:1, 2:1, 5:1, 10:1. In contrast to CB T cells, CB CD19-CAR T cells showed strong lysis function during coculture with SUDHL-4 cells (*P* < 0.05, n = 3; **Figures 3A, B**). We observed cytotoxicity towards SUDHL-4 cells but not DB cells, which was mirrored by the anti-tumor activity post antigen stimulation (*P* < 0.05, n = 3; **Figures 3A, B**). While the outgrowth of SUDHL-4 was not affected by the dose of CB CD19-CAR T cells, the low amount (E: T of 1:1) of CB CD19-CAR T cells still led to anti-tumor activity against CB CD19-CAR T cells. CB CD19-CAR T cells cocultured with SUDHL-4 cells also showed significantly higher cytokine secretion, including IL-2, IL-4, IL-6, IL-10, TNF-α, and IFN-γ (**Figure 3C** and **Figure S1B**), further demonstrating the CD19-dependent cytotoxicity of CB CD19-CAR T cells.

To evaluate the anti-lymphoma activity of CB CD19-CAR T cells *in vivo*, we established a murine xenogeneic model using SUDHL-4 cells. Subcutaneous injection of SUDHL-4 cells into the backs of BALB/c-nu mice allowed the tumor to expand. Following confirmation of tumor engraftment on day 9, animals received CB CD19-CAR T cells or CB T cells on day 10. The growth of tumors and their weight were followed in three groups (**Figures 3D-F**). CB CD19-CAR T cells were able to control the growth of tumor compared with CB T cells and untreated group, the representative images and data from n = 4 mice per group (**Figure 3D**). No significant decrease in mouse body weight or other toxicity signs was observed in any treatments, including CB T cells and CB CD19-CAR T cells, suggesting little systemic toxicity with good tolerability (**Figure 3F**). These results support the results of our *in vitro* study and indicate that CB CD19-CAR T cells effectively inhibit tumor growth in a DLBCL model.

### Changes in genes expression of CB CD19-CAR T cells following coculture

**Figure 4:**
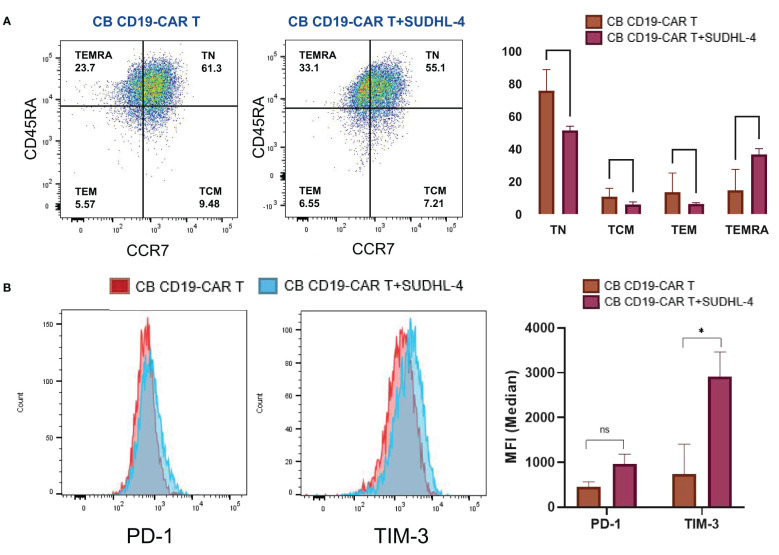
Phenotypic and numeric changes of the coculture of CB CD19-CAR T with the DLBCL cells. **(A)** Representative flow cytometric plots of the CB CD19-CAR T after coculture of SUDHL-4 cells (left panel). Data show mean ± SEM (right panel). **(B)** Representative flow cytometry histogram and representative histogram of PD-1 and TIM-3 expression in CB CD19-CAR T alone and after coculture with SUDHL-4 cells (left panel). Bar graphs show mean ± SEM (right panel). TN, Naive T cell; TCM, Central memory T cell; TEM, effector memory T cell; TEMRA, Terminal effector T cell; PD-1, programmed cell death protein-1; TIM-3, T cell immunoglobulin and mucin domain-containing protein 3; SEM, standard error of means; ns, non-significant; *P < 0.05; **P < 0.01.

We analyzed the changes after CB CD19-CAR T cell interaction with tumor cells. After 48h of coculture, CB CD19-CAR T cells showed loss of the CCR-7 phenotype and naïve T cells converted them into terminally differentiated effector memory cells re-expressing CD45RA (T_EMRA_) T cells; the formerly naïve CB T cell population promptly differentiated into an effector cell (**Figure 4A**). The upregulation of immune checkpoint proteins might limit the anti-tumor activity causing resistance of immune cell-mediated therapy. Among them, programmed cell death protein-1 (PD-1) and T cell immunoglobulin and mucin domain-containing protein 3 (TIM-3) have recently received increased attention for playing a critical role in inhibition of T cell proliferation and function. Therefore, we investigated changes in the expression of PD-1 and TIM-3 on the surface of CB CD19-CAR T cells. As shown by our flow cytometry results (**Figure 4B**), mean TIM-3 expression was significantly higher in CB CD19-CAR T cells after coculture with SUDHL-4 cells. PD-1 expression levels were not statistically significant. CB-derived CAR T cells showed elevated immune checkpoints after coculture with SUDHL-4, which might hinder the ability of CB CAR T cells to expand and act continuously *in vivo*.

**Figure 5:**
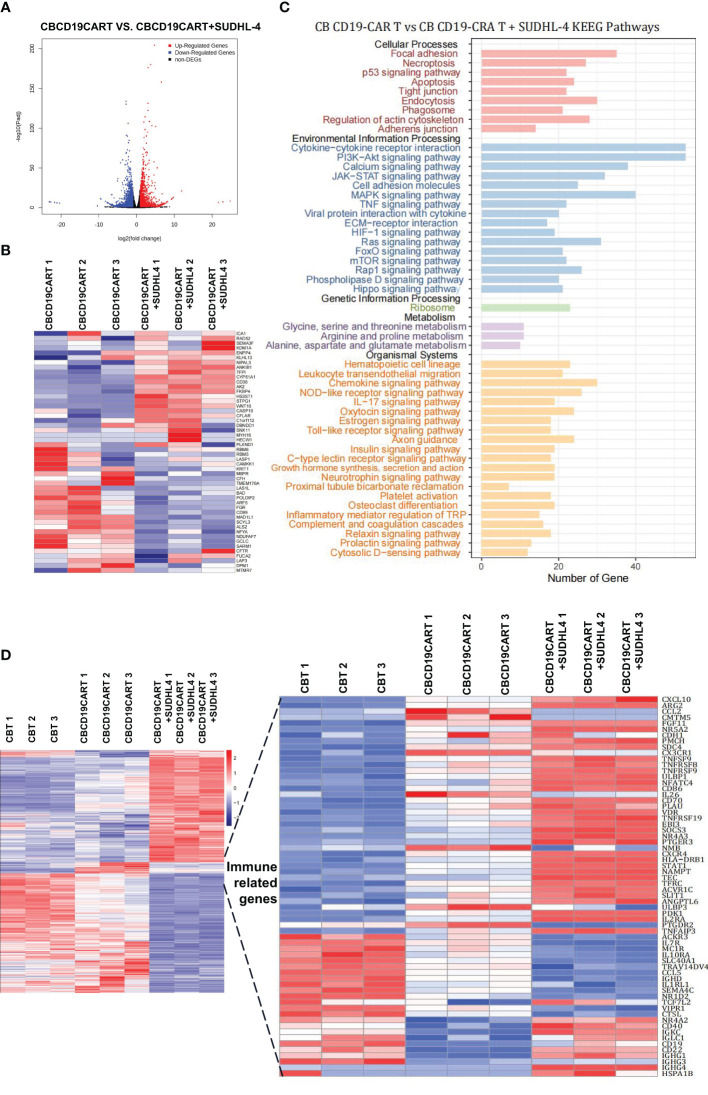
RNA-seq analysis of the coculture of CB CD19-CAR T with the DLBCL cells. **(A)** The distribution of DEGs between CB CD19-CAR T alone and after coculture with SUDHL-4 cells. **(B)** Heatmap of top 50 DEGs. **(C)** KEGG pathways of DEGs between CB CD19-CAR T alone and after coculture with SUDHL-4 cells. **(D)** Heatmap for immune-related gene sets of CB T group, CB CD19-CAR T alone group and after coculture with SUDHL-4 cells group. DGEs, differentially expressed genes.

To elucidate which gene is responsible for these changes, we analyzed the RNA-seq data of CB CD19-CAR T cells cocultured with or without SUDHL-4 cells. The resulting SUDHL-4 cells were cultured for 48 h when CB CD19-CAR T cells were selected using magnetic beads. We identified 3331 DEGs, 1584 upregulated and 1747 downregulated, in the two comparisons (**Figure 5A**). The top 50 DEGs following coculture was listed in **Figure 5B**. KEGG analysis of the top DEGs showed that immune-related gene pathways were mainly altered following coculture. A functional enrichment analysis in all two comparisons showed that most of the KEGG pathways were signal “focal adhesion” “cytokine-cytokine receptor interaction,” and “chemokine signaling pathway,” which are associated with recognition or killing by CAR T cells binding to tumor cells (**Figure 5C**).

Next, we analyzed DEGs associated with immunity between CB T subsets, CB CD19-CAR T subsets, and CB CD19-CAR T/SUDHL-4 coculture subsets in our combined dataset. Hierarchical clustering of 65 immune-related genes led to the identification of chemokines/chemokine receptors (*CXCL10, CCL2, CX3CR1, CXCR4*, and *CCL5*), costimulation (*TNFRSFs* gene families), exhaustion- (*NR4As* gene families), and memory-associated genes (*IL7R* and *IL2RA*) (**Figure 5D**). Coculture of CB CD19-CAR T cells with SUDHL-4 cells significantly upregulated canonical exhaustion-associated genes (*NR4A3*), costimulation genes (*TNFSF9, TNFRSF8*, and *TNFRSF9*) and *STAT1*, and downregulated memory-associated genes (*IL7R, CXCL10*, and *CXCR4*) compared to coculture with CB T cells. Notably, we found that genes associated with ferroptosis (*TFRC* and *SLC40A1*) in CB CD19-CAR T/SUDHL-4 coculture were more likely to be differentially expressed compared to CB T and CB CD19-CAR T. Here, it may indicate the involvement of ferroptosis in CB CD19-CAR T cell death (**Figure 5D**).

## Discussion

CAR T cell therapy has been presented as a second or even first-line treatment in patients with R/R LBCL (23–25). The ‘off-the-shelf’ product is under intense investigation to enable higher and broader availability of CAR T therapy. Several studies have shown that CB, a lesser-used source of CAR T cells, is an effective source of cancer immunotherapy (22, 26). For example, studies have used primary cells from CB to culture-specific T cells that target acute myeloid leukemia and ALL (27, 28). The activity of CB-derived CAR T cells has also been confirmed in ALL cell lines and mouse models (22). Additionally, CAR-NK cells from CB cells have been safely administered without complete HLA matching and showed practical anti-tumor effects in NHL. Considering the unique characteristics of CB, we designed a study on the application of CB CD19-CAR T cells in CD19+ DLBCL. CB CD19-CAR T cells displayed cytotoxicity targeting the CD19+ T cell lymphoma cell line BV173 and CD19+ DLBCL cell line SUDHL-4, triggered secretion of multiple cytokines in coculture assays, and limited tumor growth in a mouse model. Gene expression profiles confirmed increased chemokines/chemokine receptors and exhaustion genes in CB CD19-CAR T cells upon challenge with tumor cells compared to CB T cells. Our results show that CB CD19-CAR T cells are a promising therapeutic strategy for treating DLBCL.

A single dose of CB can amplify 10^8^ CAR T cells, and CB T cells have an advantage over auto-CAR T cells because of insufficient T cells in post-chemotherapy patients. T cells derived from CB also possess a unique antigen-naïve status (29). There is ample evidence that demonstrates different subsets of naïve T cells play distinct roles in immunity (30) and that the stemness of anti-tumor T cells can increase the potential of immunotherapy (31). CAR T cells constructed with different costimulatory domains show different features. We built CB CD19-CAR T cells using 4-1BB as a costimulatory molecule, as CARs confer longer persistence in the presence of 4-1BB (32). Additionally, 4-1BB-based T cells tend to behave like central memory-like T cells, improving mitochondrial and expiratory capability and fatty acid metabolism (33). Moreover, we argue that CB CD19-CAR T cells could specifically recognize and kill the CD19+ ALL cell line BV173 and DLBCL cell line SUDHL-4 in an antigen-specific manner *in vitro* and control tumor progression *in vivo*. Overall, we determined that CB CD19-CAR T cells show specific cytotoxicity and simultaneous cytokine production can effectively eliminate CD19+ DLBCL cells.

PBMCs-naïve T cells cause severe GVHD in murine models (34). However, T cells derived from CB were transformed into CAR T cells after transfection with a surface antigen specific CAR because these cells lack the CD3/TCRab complex; therefore, their responses are not HLA-restricted (35), which is a characteristic of the placenta. Different from all other tissue cells, extravillous cytotrophoblast cells in the placenta express only HLA-C, HLA-E, and HLA-G, and syncytiotrophoblast cells are HLA-negative (36); these potential features result in minimal risk of GVHD (37). Furthermore, this implies an additional reason for the decreased risk of GVHD. The reactivity of CB T cells is reduced by impaired nuclear factor of activated T cell signaling (38). We observed a weight reduction in the CB CD19-CAR T cell group after treatment compared with the control group, but it increased again after a week. No diarrhea, rash, or jaundice, which are common symptoms of GVHD, were observed during the observation period. We concluded that CB CD19-CAR T cells were associated with minimal GVHD.

No response and secondary resistance after CAR T cell therapy are clinical conundrums in the CAR T cell therapy era (39). CAR T cell expansion and persistence are essential components for CAR T efficacy, patients achieving CR, and preventing relapse. Defining phenotypic and functional changes in CAR T cells is paramount for developing practical CAR-T strategies (40). Our study also elucidated that after coculture with DLBCL cell lines, CB CD19-CAR T cells show significantly upregulated *TNFSF9, TNFRSF8, TNFRSF9*, and *STAT1* compared with CB T and CB CD19-CAR T cells. Several TNFR family members participate in sustaining T cell responses after T cell activation (41). Another study demonstrated that the STAT1 pathway defends T cells from NK cell-mediated eradication involved in T cell survival (42). CB CD19-CAR T cells may be activated by naïve CB T cells to initiate the STAT1 signaling pathway and TNK pathway and release cytokines to play an effector role. However, we also found that *NR4As* gene families were upregulated, and IL7R was downregulated in the coculture group compared with CB CD19-CAR T alone. *NR4As* genes play an essential role in T cell dysfunction and cause CAR T cells to enter an exhausted or dysfunctional state in solid tumors (43, 44). Previous reports have shown that *IL7R* blocks the development of T cells, and patients with *IL7R-*inactivating mutations present with severe combined immunodeficiency (45, 46). Short persistence and early exhaustion of T cells are significant limitations to immunotherapy efficacy and its broad application (47, 48). Thus, targeting *IL7R* and *NR4A* is a promising CAR T cell therapy strategy. Many strategies, such as designing CB-derived CAR T cells with specificity to immunodeficiency genes and virus-specific antigens (49), must be explored to address these problems. Nevertheless, our work addresses a significant barrier to the progress of this emerging class of therapeutic agents. These possibilities will be examined in the future to develop CAR T therapy.

In conclusion, we generated CB CD19-CAR T cells and confirmed their anti-tumor activity against DLBCL cells. We also studied the underlying cellular pathways in CB CAR-T cells and explored their exhaustion mechanisms. The development of CB CAR T cells as an ‘off-the-shelf’ CAR T cell readily available for patients with R/R LBCL in an affordable and timely manner would significantly get patients close to these therapeutics. Our trial results could help inform patients who require immunotherapy of more excellent choices.

## Data availability statement

The datasets presented in this study can be found in online repositories. The names of the repository/repositories and accession number(s) can be found below: BioProject *via* accession ID: PRJNA924756 https://dataview.ncbi.nim.nih.gov/object/PRJNA924756?reviewer=ktch835c5g69b09tia6qphv13f.

## Ethics statement

The animal study was reviewed and approved by Department of Hematology, The Second Affiliated Hospital of NanChang University.

## Author contributions

TY and LY designed the study; TY, CL, and LY performed the experiments and analyzed the data, TY and LY wrote the manuscript. All authors contributed to the article and approved the submitted version.
