# Supplementary figures and images for: Cord blood-derived CD19-specific chimeric antigen receptor T cells: an off-the-shelf promising therapeutic option for treatment of diffuse large B-cell lymphoma
Source: Front Immunol. 2023 Jun 27;14:1139482. doi: 10.3389/fimmu.2023.1139482 (PMC10338183; doi:10.3389/fimmu.2023.1139482)

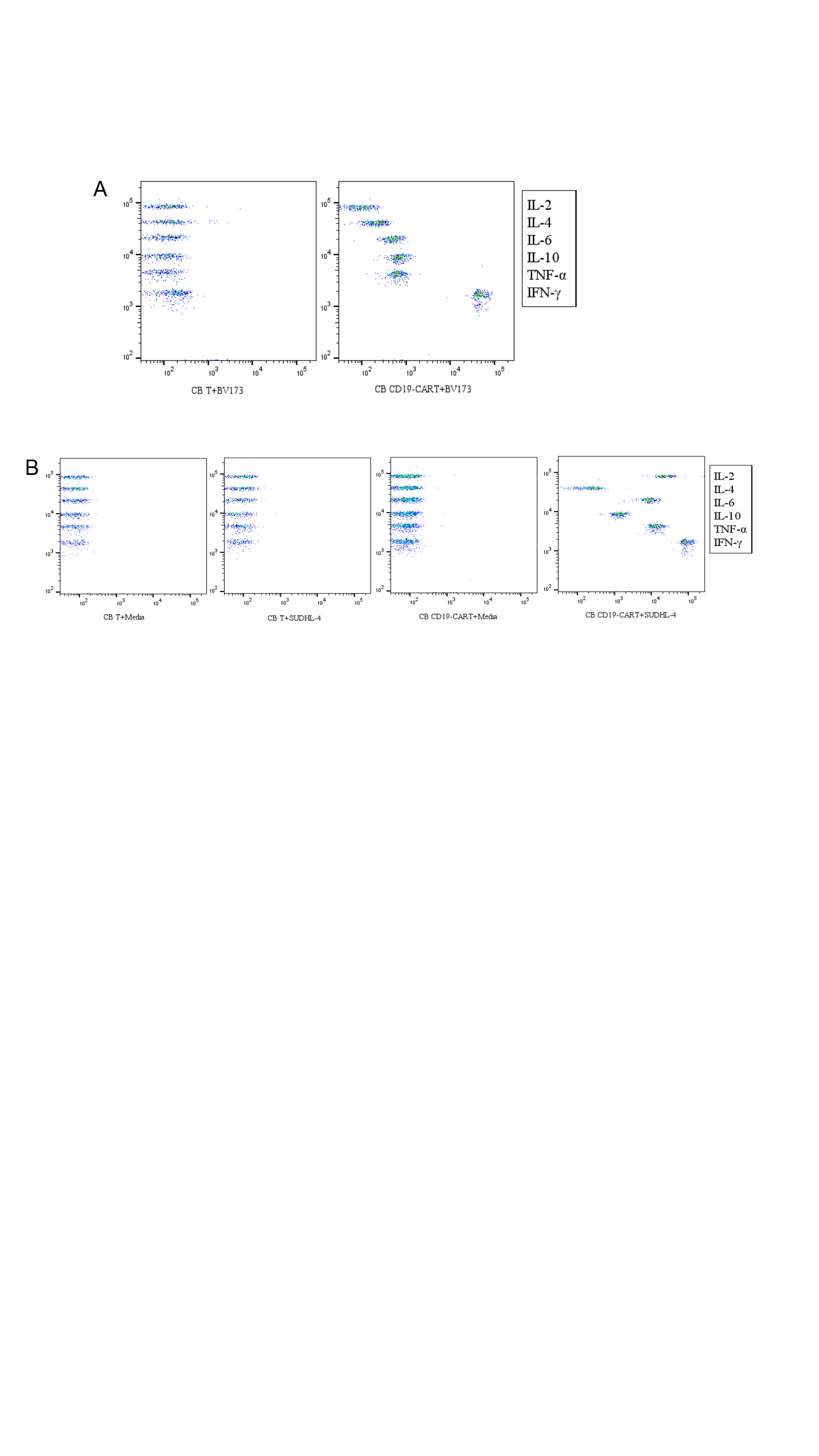

Supplement: Supplementary file 1 [file Image_1.tif]
